# Carcinome épidermoïde de la vésicule biliaire compliquant une dilatation kystique de la voie biliaire principale (VBP) et du canal cystique: à propos d´un cas

**DOI:** 10.11604/pamj.2021.38.144.22684

**Published:** 2021-02-09

**Authors:** Mohamed Boudou, Rachid Jabi, Christine Kora, Achraf Miry, Imane Kamaoui, Mohammed Bouziane

**Affiliations:** 1 Service de Chirurgie Viscérale et Oncologie Digestive A, Centre Hospitalier Universitaire Mohammed VI, Oujda, Maroc,; 2 Service de Radiologie, Centre Hospitalier Universitaire Mohammed VI, Oujda, Maroc,; 3 Service de l´Anatomopathologie, Centre Hospitalier Universitaire Mohammed VI, Oujda, Maroc

**Keywords:** Carcinome épidermoïde, vésicule biliaire, dilatation kystique, voies biliaires, à propos d´un cas, Squamous cell carcinoma, gallbladder, cystic dilation, bile ducts, case report

## Abstract

La maladie kystique congénitale de l´arbre biliaire est considérée parmi les facteurs de risque connus du cancer de la vésicule biliaire. Nous rapportons le cas d´un carcinome épidermoïde de la voie biliaire (VB) compliquant une dilatation kystique des voies biliaires chez une femme de 54 ans qui a été hospitalisée pour pancréatite biliaire avec un scanner abdominal montrant un épaississement nodulaire du fond vésiculaire et une dilatation fusiforme du canal cystique et de la voie biliaire principale (VBP) avec une lésion de la queue du pancréas évoquant en premier lieu un cystadénome mucineux. Une cholécystectomie élargie au lit vésiculaire emportant la voie biliaire distale, anastomose cholédoco-duodénal avec une spléno-pancréatectomie caudale + drainage étaient réalisées. L´histopathologie de la masse de la vésicule biliaire a révélé un carcinome épidermoïde modérément différencié invasif sans infiltration du parenchyme hépatique. La patiente a commencé une chimiothérapie adjuvante. Notre patiente ne présentait pas les symptômes typiques du cancer de la vésicule biliaire et la radiologie était nécessaire pour son diagnostic. La chirurgie reste la meilleure option thérapeutique pour le cancer de la vésicule biliaire à un stade précoce, tandis que la chimio-radiation adjuvante peut également être bénéfique. La cholécystectomie et la résection de la dilatation kystique de la voie biliaire chez les patients à haut risque constituent le moyen de prévention le plus performant.

## Abstract (en)

Congenital cystic disease of the biliary tree is a known risk factor for gallbladder cancer. We here report a case of squamous cell carcinoma of the bile duct (BD) complicating a cystic dilatation of the bile ducts in a 54-year-old woman hospitalized for biliary pancreatitis. Abdominal scanner showed nodular thickening of the fundus of the gallbladder and fusiform dilation of the cystic duct and the main bile duct (VBP) with lesion of the tail of the pancreas, initially suggesting mucinous cystadenoma. Extended cholecystectomy involving the gallbladder fossa with resection of the distal biliary tract, choledocoduodenal anastomosis with caudal splenopancreatectomy + drainage were performed. Histopathological examination of the gallbladder mass revealed moderately differentiated invasive squamous cell carcinoma without infiltration of the hepatic parenchyma. The patient underwent adjuvant chemotherapy. The patient did not have the common symptoms of gallbladder cancer. Then radiology was necessary to make a diagnosis. Surgery is the best therapeutic option for early-stage gallbladder cancer, but adjuvant chemo-radiation may also be useful in treating these patients. Cholecystectomy with resection of cystic dilatation of the bile duct in high-risk patients are the most effective means of prevention.

## Introduction

Le cancer primitif de la vésicule biliaire représente le cinquième cancer des tumeurs malignes digestives [1]. Histologiquement, les adénocarcinomes (83%) sont les plus fréquents, Le carcinome épidermoïde est une tumeur très rare représente moins de 2% des tumeurs malignes de la vésicule [1,2]. Les facteurs de risque du cancer de la vésicule biliaire et des voies biliaires en général sont les causes d´inflammation biliaire chronique. Parmi ces facteurs de risque connus, on trouve la dilatation kystique de la voie biliaire (DKVB) [3]. Nous présentons un cas de carcinome épidermoïde primaire de la vésicule biliaire compliquant une dilatation kystique du canal cystique et du cholédoque et nous voudrions insister sur le risque de cancer dans les formes découvertes à l´âge adulte, l´histogénèse ainsi que la prise en charge thérapeutique de cette entité rare.

## Patient et observation

**Figure 1:**
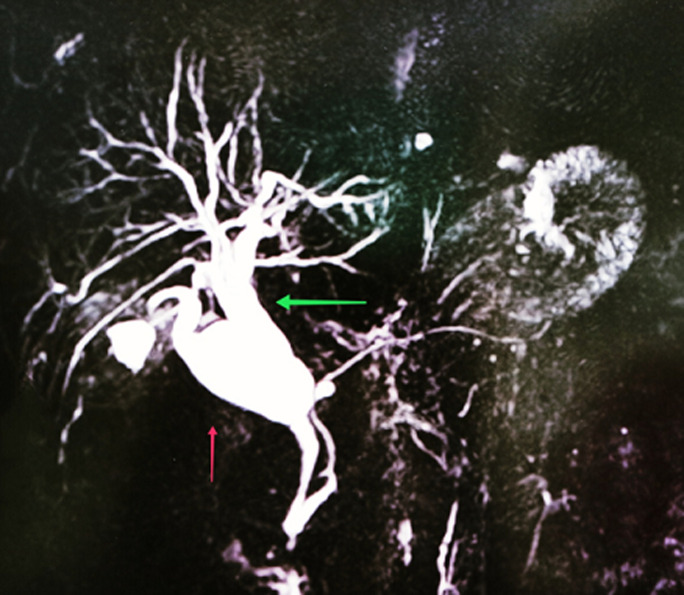
séquence 3D BILI-IRM montrant la dilatation du cystique (flèche rouge) et du cholédoque (flèche verte)

**Figure 2:**
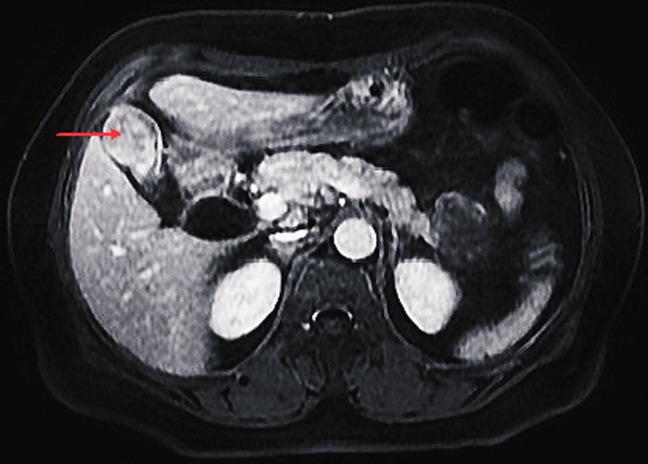
coupe axiale montrant le processus tumoral après injection de gadolinium (flèche rouge)

Patiente âgée de 54 ans, ayant comme antécédent une thyroïdectomie totale pour un carcinome papillaire de la thyroïde sous levothyrox 125 µg. Elle était hospitalisée pour prise en charge d´une pancréatite biliaire stade B. A l´examen physique la patiente était stable sur le plan hémodynamique et respiratoire, apyrétique, avec une sensibilité à la palpation de l´épigastre et de l´hypochondre droit. Biologiquement, on notait une cytolyse (ASAT: 619 UI/L; ALAT: 527 UI/L) avec un cholestase (BT/: 26 mg/l BD: 19mg/l) et une hyperlipasémie (518 UI/l: sept fois la normale). Radiologiquement, la patiente a bénéficié d´une tomodensitométrie thoraco-abdomino-pelvienne avec injection de produit de contraste à objectivé un épaississement nodulaire non circonférentiel du fond vésiculaire mesurant 33x24mm, faisant évoquer en premier une origine tumorale, à confronter aux données histologiques. Une pancréatite œdémateuse interstitielle stade B de Balthazar avec un Balthazar modifié à 1, avec lésion kystique de la queue du pancréas pouvant être en rapport avec un cystadénome mucineux. Une dilatation kystique du canal cystique avec dilatation des VBIH et de la VBP à 15 mm sans obstacle visible (à confronter aux données de l´IRM) Une BILI-IRM a montré un épaississement nodulaire du fond vésiculaire, une dilatation fusiforme du canal cystique et de la VBP, avec une trifurcation biliaire (Figure 1, Figure 2) avec une lésion de la queue du pancréas uni loculée faisant évoque en premier lieu un cystadénome mucineux.

**Figure 3:**
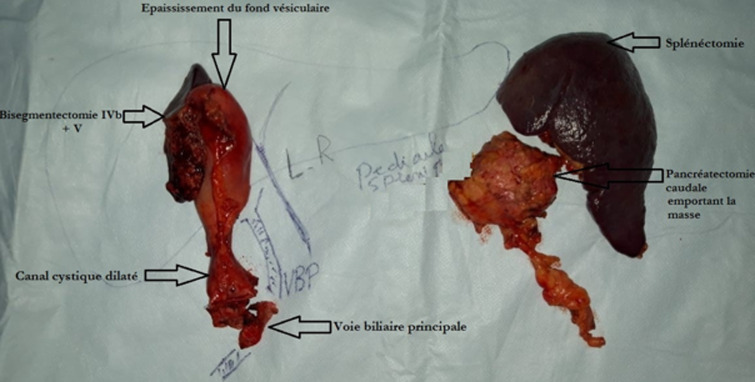
image opératoire d’une pièce de résection de la vésicule biliaire élargie au lit vésiculaire emportant la voie biliaire distale avec une spléno-pancréatectomie caudale

**Figure 4:**
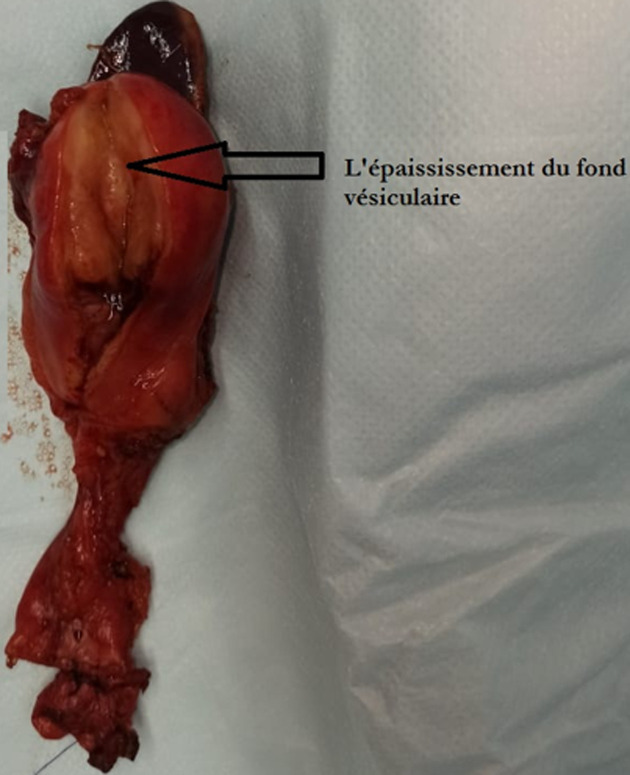
image opératoire d’une pièce de résection de la vésicule biliaire montrant l’épaississement du fond vésiculaire

**Figure 5:**
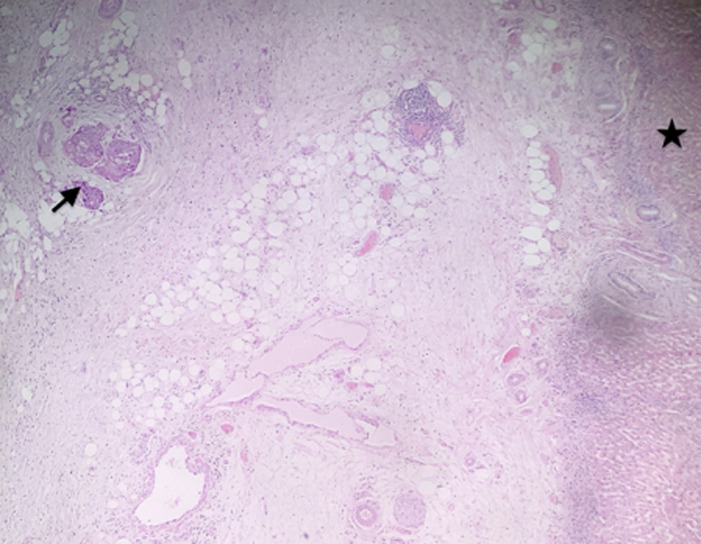
microphotographie montrant la prolifération carcinomateuse (flèche) avec un parenchyme hépatique visible, qui est non envahi (étoile) (HE; 40X)

**Figure 6:**
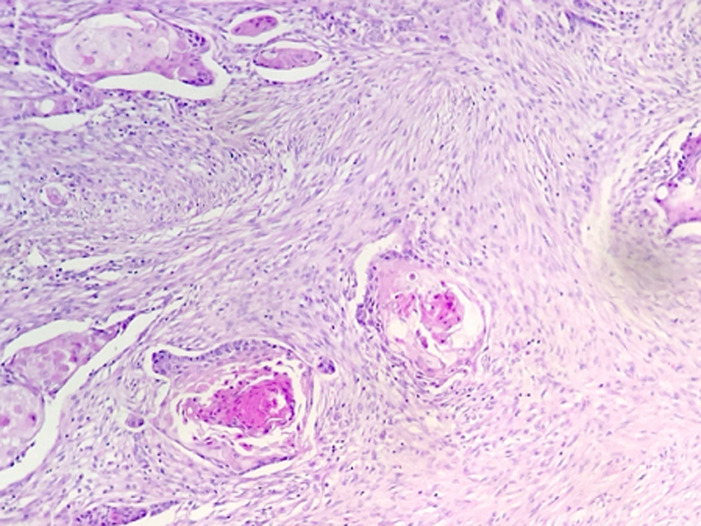
microphotographie montrant que la prolifération est faite de nids et de massifs de cellules éosinophiles de grande taille; de nombreux globes cornés sont visibles (HE; 200X)

La patiente a été opérée par voie bi sous costale, l´exploration per opératoire, montrait une tumeur du fond de la vésicule biliaire avec une dilatation kystique du canal cystique et du cholédoque et une lésion kystique de la queux du pancréas après ouverture de l´arrière-cavité des épiploons. Une cholécystectomie élargie au lit vésiculaire emportant la voie biliaire distale + anastomose cholédoco-duodénale termino-latéral avec une spléno-pancréatectomie caudale + drainage étaient réalisées avec des suites immédiates simples (Figure 3, Figure 4). L´examen anatomopathologique de la pièce était en faveur d´un carcinome épidermoïde bien différencié de la paroi vésiculaire classé T2 avec un faux kyste du pancréas (Figure 5, Figure 6). Absence d´emboles vasculaires ni engainement péri nerveux. Cette tumeur était classée stade p T2 selon la classification TNM. Une chimiothérapie adjuvante à base de Capecitabine était entamée avec une bonne évolution clinique.

## Discussion

La maladie kystique congénitale de l´arbre biliaire c´est une maladie bénigne, mais leur présence est associée à des complications graves, y compris un carcinome biliaire [4]. Elle est principalement présente chez les enfants. Elles sont quatre fois plus fréquentes chez les femmes. Dans les pays développés, seuls 20 à 30% des patients sont diagnostiqué à l´âge adulte [4]. Todani *et al*. a proposé une classification modifiée, actuellement plus largement employée car tient compte de l´état des voies biliaires intra-hépatiques [5]. Dernièrement, il est bien établi que la DKVB est la conséquence d´une anomalie de la jonction bilio-pancréatique. Cette malformation de fusion entre les canaux biliaire et pancréatique est caractérisée par trois critères: a) canal commun anormalement long (>15mm); b) une jonction extra duodénale des deux canaux à distance des sphincters; c) un angle de raccordement supérieur à 30° [6,7]. Cette anomalie est retrouvée dans 70% des cas dans le type Ia et dans 30% des cas dans le type Ic [8]. Cette anomalie de la jonction bilio-pancréatique est incriminée aussi dans la dégénérescence des segments biliaires dilatés et de la vésicule biliaire [9]. Les patients portent un risque de cancer des voies biliaires, avec une augmentation de l´incidence avec un âge <1% chez les patients de moins de 10 ans, 15% chez les patients âgés de plus de 20 ans, et jusqu´à 35% chez les patients >60 ans [10]. Soixante-dix pourcent (70%) des tumeurs sont situé dans les voies biliaires et 30% dans la vésicule biliaire [10]. La dérivation kysto-duodénale ou kysto-jéjunale majore le risque de transformation maligne [11]. Car le reflux de liquide intestinal dans la voie biliaire accélère les phénomènes d´ulcération, régénération épithéliale métaplasique et par conséquent l´évolution vers le cancer [12]. L´adénocarcinome est le type histologique le plus fréquent (90% des cas), le carcinome épidermoïde reste une entité histologique qui ne représente que moins de 2% des cancers de la VB, certaines hypothèses suggèrent une métaplasie malpighiacée de l´épithélium de la vésicule biliaire. D´autres proposent une transformation maligne à partir d´un épithélium malpighien hétérotopique congénital au niveau de vésicule biliaire. L´hypothèse la plus vraisemblable reste une métaplasie malpighienne à partir d´un adénocarcinome préexistant, donnant naissance à un carcinome adénosquameux. Par la suite, la métaplasie malpighienne évolue rapidement et remplace la composante glandulaire pour donner un carcinome épidermoïde. Sur le plan clinique, aucun signe clinique n´est pathognomonique au cancer développé sur DKVB [13]. La tumeur siégeant dans la zone dilatée ne devient que tardivement obstructive [13] et lorsque l´ictère cholestatique apparaît, la lésion est déjà très évoluée.

Une hépatomégalie ou masse palpable avec altération de l´état générale témoignent souvent d´une extension dépassée. Le diagnostic radiologique des DKVB, chez l´adulte, ne devrait pas poser de difficulté dans la majorité des cas [11]. Le diagnostic initial est basé sur l´échographie abdominale, mais la bili-IRM permet de préciser le type de dilatation, l´existence d´anomalie de la jonction bilio-pancréatique, et la présence d´un calcul intrakystique ou une lésion tissulaire pariétale pouvant témoigner une dégénérescence. La cholangiopancréatographie rétrograde endoscopique (CPRE) a un double intérêt diagnostique (caractère communicant du kyste du cholédoque) et thérapeutique (extraction d´éventuels calculs intrakystiques) mais elle est supplantée par la cholangio-wirsungo-IRM [14-16]. La tomodensitométrie avec injection de produit de contraste permet de préciser la nature tissulaire d´un nodule pariétal et de détecter les toutes petites lésions [17]. Le traitement de choix est l´exérèse qui prévient la dégénérescence de la paroi kystique et des voies biliaires. Les dérivations kystodigestives sont à proscrire [16]. L´exérèse de la dilatation kystique et de la vésicule biliaire avec une anastomose hépatico-jéjunale est actuellement la plus préférée et ce quel que soit le type de dilatation kystique. La résection de la convergence doit être d´autant plus étendue que celle-ci est kystique. Lorsqu´il existe des anomalies intra-hépatiques uni lobaires associées, une résection hépatique partielle doit être envisagée [7,11,16]. Concernant notre cas Le traitement curatif d´un carcinome épidermoïde ne diffère pas des autres carcinomes et dépend de l´extension locorégionale. Il demeure basé sur la résection chirurgicale, étendue sur le lit vésiculaire. Malgré la faible extension lymphatique, qui est une caractéristique du carcinome épidermoïde, un curage ganglionnaire est recommandé [18]. Seule la résection R0 peut améliorer le pronostic de ces carcinomes épidermoïdes. Comme pour les adénocarcinomes vésiculaires, la réalisation d´une cholécystectomie chez les patients à haut risque (calcul de plus de 3cm, polype>1cm, reflux pancréatico-biliaire, vésicule en porcelaine, adénomyomateuse segmentaire, cholecystite xanthogranulomateuse) reste encore le moyen de prévention le plus performant.

## Conclusion

Le pronostic d´un cancer de la vésicule biliaire quel que soit sa nature histologique est sombre, la résection d´un carcinome épidermoïde de la vésicule biliaire est justifié seulement si la résection potentiellement curative (R0) est faisable. Le diagnostic précoce, la résection la plus complète possible de la dilatation kystique de la voie biliaire et la cholécystectomie prophylactique restent les seuls moyens permettant d´améliorer le pronostic.
